# Can playing Dungeons and Dragons be good for you? A registered exploratory pilot programme using offline tabletop role-playing games to mitigate social anxiety and reduce problematic involvement in multiplayer online video games

**DOI:** 10.1098/rsos.250273

**Published:** 2025-04-09

**Authors:** Joël Billieux, Loïs Fournier, Lucien Rochat, Iliyana Georgieva, Charlotte Eben, Marc Malmdorf Andersen, Daniel L. King, Olivier Simon, Yasser Khazaal, Andreas Lieberoth, Jonathan Bloch

**Affiliations:** ^1^ Institute of Psychology, University of Lausanne, Lausanne, Switzerland; ^2^ Center for Excessive Gambling, Addiction Medicine, Lausanne University Hospital (CHUV), Lausanne, Switzerland; ^3^ Specialized Facility in Behavioral Addiction ReConnecte, Department of Mental Health and Psychiatry, Geneva University Hospitals (HUG), Geneva, Switzerland; ^4^ Faculty of Psychology, UniDistance, Brig, Switzerland; ^5^ Centre for Gambling Research at UBC, Department of Psychology, University of British Columbia, Vancouver, Canada; ^6^ Biological Psychology, Department of Psychology, University of Cologne, Cologne, Germany; ^7^ Interacting Minds Centre, School of Culture and Society, Aarhus University, Aarhus, Denmark; ^8^ College of Education, Psychology and Social Work, Flinders University, Adelaide, Australia; ^9^ Department of Psychiatry and Addictology, Faculty of Medicine, University of Montreal, Montreal, Canada

**Keywords:** gaming disorder, MMORPG, problematic gaming, registered report, role-playing game, social anxiety treatment, TTRPG

## Abstract

Gamers with poor self-concept, high social anxiety and high loneliness are more at risk of problematic involvement in video games, such as massively multiplayer online role-playing games. There is a research gap concerning treatment approaches to cater to socially anxious gamers with problematic patterns of gaming involvement. This registered exploratory pilot programme tests the feasibility and initial effect of a structured protocol in which socially anxious online gamers are exposed to real-life social interactions while playing an offline tabletop role-playing game (TTRPG). Our structured protocol lasted 10 weeks and involved 10 sessions organized into three modules in which participants played a TTRPG inspired by the game ‘Dungeons and Dragons’. Each module deployed a role-playing scenario designed to challenge the players in game terms and involve them in a story based on maturing relationships with other characters and solving challenges by social means and investigation. Our study used a quasi-experimental multiple single-case design with a three-week baseline across groups (four groups of five gamers with sub-clinical problematic video game use and social anxiety) and a three-month follow-up. Primary outcomes were time spent gaming, gaming disorder symptoms and social anxiety symptoms. Secondary outcomes were assertiveness/social skills, self-concepts and perceived loneliness. In terms of feasibility, we observed that most participants completed the programme (two of the 20 participants dropped out) and were involved in terms of participation and weekly psychometric assessments. Moreover, participants were largely able to attain the progressively more difficult objectives implemented in the TTRPG programme. Multiple single-case analyses showed that most participants benefited from the intervention through a reduction in social anxiety symptoms and problematic gaming symptoms, although to varying degrees. Some participants also reduced their gaming time or presented with reduced perceived loneliness. Assertiveness and self-concepts were not improved. This pilot study shows that a TTRPG intervention approach is feasible and may be used to reduce social anxiety and gaming disorder symptoms. The present programme must now be tested with clinical participants.

## 1. Introduction

Video games are one of the most popular leisure activities worldwide. The number of gamers is expected to reach 3.6 billion players in 2025 [1]. Although most players enjoy gaming as a recreational activity, some individuals report uncontrolled and excessive gaming that has negative consequences (e.g. addiction symptoms, health consequences, conflicts with family) and that is functionally impairing [2,3]. In 2019, gaming disorder was included as a mental disorder in the 11th edition of the *International Classification of Diseases* [4], and its worldwide prevalence is estimated to be around 1–2% [5]. Psychological models posit that over-involvement in video games and virtual worlds may serve to fulfil needs unachieved in real life through a compensatory process [6–8]. A systematic review on maladaptive player–game relationships reported that gamers who report diminished self-concept (e.g. poor self-esteem), high social anxiety and high loneliness are at risk of being problematically involved in video games that have role-playing and social interaction components, such as massively multiplayer online role-playing games (MMORPGs) [9].

In Switzerland, the country in which the present study took place, data from an outpatient clinic that specializes in the treatment of ‘behavioural addictions’ (also called ‘non-substance-related addictive behaviours’) reported that over-involvement in MMORPGs was a frequent treatment motive and that social phobia was the most frequent comorbid psychiatric disorder experienced among these gamers [10]. Despite gaming-related harms increasingly being recognized as a public health issue [11], we remain in need of evidence-based psychological interventions to support those seeking help with problematic video game use [12–14]. Crucially, an important gap in research concerns a treatment approach adapted to socially anxious gamers.

### 1.1. Massively multiplayer online role-playing games

In MMORPGs, players create a character that evolves within a digital virtual world where they can interact with thousands of other players and the game environment. Players assume the roles of fictional characters who act and evolve in virtual worlds often inspired by heroic fantasies such as J.R.R. Tolkien’s saga, *The Lord of the Rings* [15]. One example is *World of Warcraft*, which is still considered one of the most iconic and representative MMORPGs and which numerous psychological studies have centred on in the last two decades (e.g. Bessière *et al.* [16], Billieux *et al.* [17]). In MMORPGs, character creation often involves components like the selection of a class (e.g. warrior, mage, rogue, priest), race (e.g. human, elf, orc) and avatar appearance (a visual representation of the character in the virtual world). The concept of progression is a central component in most MMORPGs, meaning that a player’s character will acquire new skills and powers and amass virtual currency and belongings as rewards for exploring and succeeding in missions or quests (e.g. defeating a powerful monster, finding a specific item).

Another fundamental aspect of MMORPGs is cooperative social interaction, which is often mechanically necessary for progressing and resolving quests, as well as obtaining and harnessing in-game resources to overcome challenges. When playing, it is possible to communicate easily with other players (e.g., text-based chat or audio). Players frequently group themselves in guilds (hierarchical organizations of characters with common objectives) organized both in-game and on platforms like *Discord*. Crucially for our purposes, most of these MMORPG features (e.g. character creation, progression system, teamwork) find their roots in *Dungeons and Dragons* (DnD), the first broadly available tabletop role-playing game (TTRPG) created by Gary Gygax and Dave Arneson in 1974. Since its creation, DnD has been a cornerstone of role-playing adventure games, inspiring similar games across the world, and its current popularity extends to the broader cultural *zeitgeist* (e.g. spin-off movies, references on globally popular TV shows such as *The Simpsons, The Big Bang Theory* and *Stranger Things*).

### 1.2. Tabletop role-playing games

**Figure 1.**
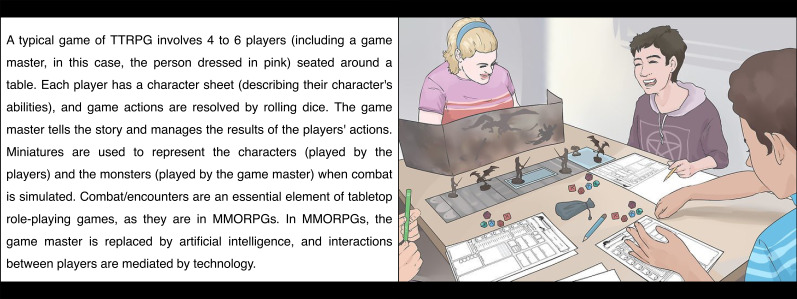
Tabletop role-playing games (TTRPGs).(Note: the illustration was retrieved from https://www.wikihow.com/Create-a-Dungeons-and-Dragons-Character.)

In TTRPGs, a group of players (usually three to five) plays together and forms a party composed of characters involved in an adventure. Together, the players must coordinate their efforts and competencies to interact with the world they construct in their shared imagination, supported by rules, game lore and sometimes artefacts like maps, drawings or miniature models. The story and exchanges may be entirely improvised, yet they commonly follow a written scenario, known only fully by one participant taking on the role of game master, who describes events and referees how player decisions affect the world (see figure 1). Typically, players must solve problems, do detective work, tackle social issues, engage in epic battles, and gather treasure and knowledge. Through this process, the characters earn experience, gain ‘levels’ and become increasingly powerful over a series of separate gaming sessions. In this way, players often become increasingly invested in their character and develop their role in the group and the broader world of the fantasy game. Beyond these similarities, there are major differences between TTRPGs such as DnD and MMORPGs. In MMORPGs, the game master is replaced by artificial intelligence, player options are limited to what has been pre-programmed into the game engine and the interactions between players are mediated by technology and no longer take place in a classic offline (or ‘real-world’) setting.

### 1.3. Tabletop role-playing games as therapeutic tools

An emerging literature suggests that playing TTRPGs can be used in a therapeutic way to improve mental health [18–20]. It has been proposed, for example, that TTRPGs can be used as a tool in psychodynamic-oriented therapy (e.g. to unveil aspects of the self) or to promote social support and bonding in group therapy [21,22]. Of relevance to the present study, a series of recent studies have shown that TTRPGs are efficient in reducing social anxiety [23], increasing social connectedness [24] and improving social skills in children and youth with autism spectrum disorder [25]. Because social skills training and exposure to social interactions constitute evidence-based and effective treatments for social anxiety [26], it can be predicted that playing games such as DnD is likely to reduce social anxiety in problematic gamers.

### 1.4. Current study

Against this background, the current study proposes an exploratory pilot experiment that aims to test the feasibility and initial effect of a 10-week structured protocol in which persons with sub-clinical problematic video game use and a proneness towards social anxiety were actively involved in offline social interactions while playing a TTRPG with peers. In terms of feasibility, we were interested in how many sessions participants would miss and how many participants would drop out from the programme entirely, the ability of the participants to complete the weekly online psychometric assessment and the ability of the participants to understand and engage in a TTRPG as well as to succeed in the various objectives of increasing difficulty implemented in the TTRPG programme. In terms of initial efficacy, this pilot aims to explore whether our programme, which is designed to mobilize social skills by exposing the participants to socially engaging situations in real life, affects social skills (e.g. assertiveness) and self-concept (i.e. the perceived discrepancy between the ideal and actual selves, see Higgins [27] and Phillipot *et al.* [28]), reduces gaming involvement (i.e. time spent gaming) and mitigates problematic gaming, social anxiety and perceived loneliness.

We reasoned that the participants could be motivated to commit to the programme given the many similarities between TTRPGs and MMORPGs (e.g. character creation, advancement system, teamwork, heroic fantasy-based world). We capitalized on a quasi-experimental multiple single-case design to test the initial effect of our programme. A single-case design is an evaluation method that can be used to rigorously test the success of an intervention on a particular case (i.e. a specific participant). An extension of this evaluation method is the multiple single-case approach used in the current study, in which several (instead of one) cases are considered to highlight potential differences and similarities between them (e.g. factors influencing dropout, effect of the programme on primary/secondary outcomes). Evidence from multiple-case studies is generally considered more robust and reliable than single-case designs [29]. In summary, the present study represents an important proof-of-principle undertaking that could pave the way for new and creative psychological approaches to therapy delivery, particularly tailored to its target population, and may present an attractive alternative to traditional psychological treatments (e.g. individual cognitive behaviour therapy).

## 2. Methods

### 2.1. Procedure

The present study took place at the University of Lausanne (UNIL) in the French-speaking part of Switzerland. It adopted a single-case quasi-experimental design using a structured TTRPG programme for 10 weeks across four groups of five participants. Single-case methodology has unique strengths for assessing the effect of treatments and is considered a clinically relevant, rigorous and scientifically well-established alternative to traditional group comparison designs [30]. The scientific rigor of this methodology for small-scale testing of interventions like the present one has been stressed [31].

**Figure 2.**
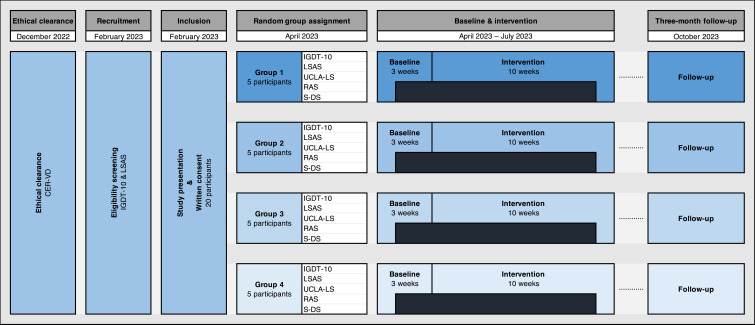
Study design and representation of the various steps of the psychological assessment. (Note: the intersections between the ‘month and year’ columns and the ‘psychometric instruments’ rows indicate which psychometric instrument was administered at which time during the baseline, intervention and three-month follow-up phases. For example, in group 1, during the baseline phase, all five psychometric instruments were administered in week 1, whereas three psychometric instruments were administered in week 2 and week 3 (i.e. IGDT-10, LSAS, UCLA-LS).)

An online survey was created to recruit participants who were eligible for the study. Eligible participants were invited to a full presentation of the study. After agreeing to participate and providing written informed consent, participants completed an initial pre-intervention psychological assessment (i.e. ‘baseline’). They then participated in the 10-week intervention. The psychological assessment was conducted online each week during the programme, and a follow-up assessment was conducted after a three-month interval to test the long-lasting psychological effects of the intervention (see figure 2). The game master managing the TTRPG sessions was part of the research team and was blinded to the results of the various psychological assessments conducted during the study. The study protocol was approved on 22 December 2022, by the Cantonal Commission on Ethics in Human Research (CER-VD; Project ID: 2022-01825). Participants were compensated 20 CHF per session (i.e. 200 CHF for 10 sessions).

All code, data, materials and preprints related to the present registered report are stored on European servers based in Frankfurt (Germany) and publicly shared under a CC-By Attribution 4.0 International license on the Open Science Framework (https://doi.org/10.17605/OSF.IO/3PGT7). The stage 1 registered report, as registered by *PCI RR* on 14 April 2023, is available on the Open Science Framework at https://doi.org/10.17605/OSF.IO/H7QAT.

### 2.2. Participants

The number of participants was determined by considering the expected dropout rate and the requirement to provide enough inter-subject replication of the intervention’s effect. TTRPGs are well suited to groups of three to five players plus one game master. We opted for five participants per group to compensate for potential dropouts. We decided that the minimum number of participants required to continue playing should be three to guarantee sufficient social exposure. It was decided that if more than two participants dropped out from the same group, the remaining participants would have been allocated (if possible) to another group.^1^ In this pilot study, dropout occurs when a participant leaves the programme permanently, regardless of the number of sessions completed. Participants who missed a session could reintegrate and continue the programme (the number of missed sessions was recorded for each participant). Participants who were enrolled in this pilot study (*n* = 20, four groups of five participants) were engaged gamers with a past or current experience of playing MMORPGs or online role-playing games (RPGs) who presented sub-clinical gaming disorder and social anxiety symptoms. The final sample size of the study was 18, as two participants dropped out (see ‘Results’ section for details). Participants who played online RPGs (e.g. *Borderlands*, *Diablo*, *Final Fantasy*)—which do not technically qualify as ‘massively’ multiplayer because they involve fewer players—were also considered eligible as those games share most features of MMORPGs (e.g. advancement mechanics, interactions between players). Furthermore, it was decided that participants with experience playing MMORPGs or RPGs were also eligible for the study even if they are currently playing other types of video games (e.g. multiplayer online battle arena). As our study is a pilot aiming to document the feasibility and initial effect of our TTRPG-based programme, we recruited participants with no clinically relevant gaming disorder or social anxiety disorder (i.e. sub-clinical cases only).

The inclusion criteria were as follows: (i) being aged 18 or older and speaking French (as playing TTRPGs and completing the various psychological assessments requires sufficient language proficiency); (ii) being a gamer with past or current experience of playing MMORPGs or online RPGs; (iii) reporting motivation to commit to playing a TTRPG for 10 consecutive weeks and to undergo weekly psychological assessment during the three-week baseline, intervention and follow-up phases; (iv) agreeing not to use a smartphone (even passively) during the TTRPG sessions (both for privacy reasons and to avoid disrupting the sessions); (v) giving informed consent by signature; (vi) endorsing at least one criterion on the Internet Gaming Disorder Test (IGDT-10; [32]) assessing gaming disorder symptoms; and (vii) having a score higher than or equal to 30 (threshold for sub-clinical social anxiety) but lower than 96 (threshold for clinical social anxiety) on the Liebowitz Social Anxiety Scale (LSAS; [33]) assessing social anxiety symptoms. Details regarding the IGDT-10 and the LSAS scales are provided in the ‘Psychological assessment’ section.

**Figure 3.**
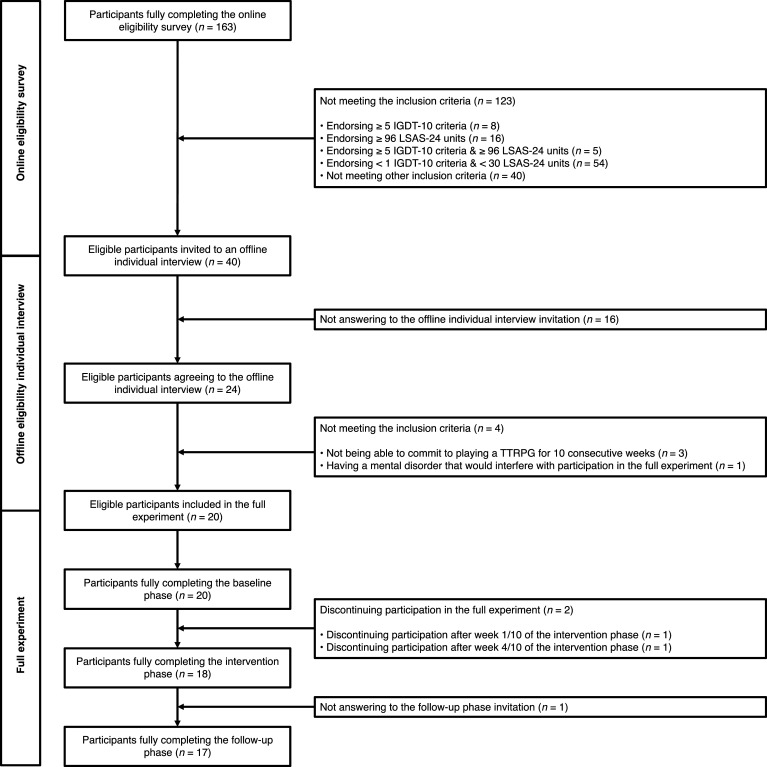
Study flowchart.

The exclusion criteria were as follows: (i) having prior experience playing TTRPGs;^2^ (ii) presenting a medical or mental condition that could interfere with the participation in the full experiment (e.g. severe cognitive impairment, physical handicap compromising commuting to the university laboratory where TTRPG sessions took place, diagnosed psychiatric condition); (iii) and presenting a possible gaming disorder.^3^ To ensure that participants did not present a clinically relevant pattern of gaming disorder, potentially eligible participants endorsing five or more gaming disorder criteria based on the IGDT-10 were redirected to the Center for Excessive Gambling at the Lausanne University Hospital (despite its name, this centre treats various kinds of behavioural addictions, including gaming disorder). At any time during the study, if a participant would have developed signs of gaming disorder, they would similarly have been addressed to the Center for Excessive Gambling.^4^ A detailed participant inclusion flowchart (indicating the number of participants included/excluded at each stage of the recruitment process) is provided in figure 3.

Eligibility was assessed via a preliminary online survey disseminated via university channels, social media (e.g. *Facebook*, *X*), a professional YouTuber and streamer (i.e. Bob Lennon), and specialized gaming forums and associations (see ‘Acknowledgements’ section for more details). A webpage was used to advertise the study (https://www.unil.ch/carla/jdr). In addition to presenting the rationale and aims of the study, this initial survey comprised a series of socio-demographic items (i.e. age, gender, education level, current professional status) and questions about gaming preferences and habits (e.g. specific game names, self-reported time spent gaming), as well as a series of psychometric questionnaires (see ‘Psychological assessment’ section for details). The first 20 participants who completed the online survey, agreed to participate in the experiment, were available to attend and play at the time proposed by the research team, and met the inclusion criteria were invited to take part in the study and were distributed into four groups (see figure 2). The distribution of participants in the various groups was done according to their availabilities (the various groups play at different times of the day and on different days of the week).^5^

Participants who did not meet the inclusion criteria received personalized feedback (see above for the specific case where a participant might present with a possible gaming disorder) and were acknowledged for their interest in the study. The recruitment process stopped as planned after securing the 20 participants (see figure 2 for details).

The age of the 20 participants ranged between 20 and 51 (*M* = 28.900, s.d. = 7.426) years, with 16 identifying as men, three as women and one as non-binary. Their weekly gaming hours ranged between 11 and 114 (*M* = 35.900, s.d. = 29.097). Their individual preferences with respect to gaming genres were varied; all 20 participants were involved in playing or had played in the past MMORPGs or online RPGs, with some also playing first-person shooter, multiplayer online battle arena, real-time strategy and other game genres. Please note that all descriptive values reported in the present paragraph were calculated using data collected in the context of the online participant eligibility screening survey (prior to participant inclusion).

### 2.3. Psychological assessment

Establishing a comprehensive and repeated baseline is crucial when applying a single-case design. For this reason, after being accepted into the study but before enrolling in the intervention, participants completed a pre-intervention psychological assessment with three measurement points (see figure 2). This three-week baseline corresponds to international standards for defining an adequate quasi-experimental single-case methodology with enough data points per phase [34].^6^ The baseline assessed gaming disorder symptoms, social anxiety symptoms and a series of psychological factors that we expected to improve with our intervention (i.e. assertiveness, self-concepts, loneliness; see below for a comprehensive description of the questionnaires used). In adopting the criteria for a single-case quasi-experimental design, we did not limit psychological assessment to the pre-intervention phase (baseline) but also performed such assessment during the intervention (i.e. after each session) and at the three-month follow-up. Self-reported gaming time and questionnaires assessing gaming disorder symptoms, social anxiety symptoms and loneliness were administered at baseline, each week during the intervention, and at follow-up. Questionnaires assessing self-concepts and assertiveness/social skills, which assess more stable psychological dimensions (i.e. less susceptible to fluctuate weekly), were administered three times: at the first baseline assessment, the end of the intervention (week 10), and the three-month follow-up. Figure 2 depicts which questionnaire was used at which step of the study for the four groups of participants.

All questionnaires used in the current study are validated in French and present adequate psychometric properties. The following sections describe the scales used to assess the abovementioned symptoms (i.e. gaming disorder and social anxiety disorders symptoms) and psychological dimensions (i.e. self-concept, assertiveness/social skills and loneliness). These sections also describe adaptations of the scales made to adjust to our multiple-single case design.

#### 2.3.1. Internet Gaming Disorder Test

The French version of the IGDT-10 assesses gaming disorder symptoms based on the *DSM-5* framework [35]. This scale was considered among the most valid, reliable and psychometrically sound screening tools to assess gaming disorder in a recent systematic review [36]. It comprises 10 items targeting the various diagnostic criteria proposed to define ‘internet gaming disorder’ in the *DSM-5* [37]. This scale assesses criteria such as loss of control, withdrawal-like manifestations (when deprived of gaming), or continued involvement in gaming despite negative consequences. Each item is scored based on frequency statements (0 = ‘never’; 1 = ‘sometimes’; 2 = ‘often’). For the eligibility screening, we followed the suggestion by Király *et al.* [35]: we considered responses ‘never’ and ‘sometimes’ as an absent criterion (0 points) and responses ‘often’ as a present criterion (1 point). As two items refer to the last *DSM-5* criterion (i.e. items 9 and 10), they were combined during the scoring procedure [32]. This coding was used to match with the categorical structure of the *DSM-5* (in which criteria are either present or absent) and identify potentially problematic gamers during the eligibility screening (endorsement of five or more criteria according to the *DSM-5* guidelines). For all statistical analyses conducted, a total score ranging from 0 to 20 was used instead to increase the variance of the scores and thus increase the likelihood of evidence change. The items referred to gaming behaviours taking place ‘over the past 12 months’ in the eligibility screening, ‘over the past week’ for the baseline and during the programme, and ‘over the past three months’ for the follow-up (see figure 2). The internal consistency (Cronbach’s alpha) of the French IDGT-10 using the same scoring method as in the current study was equal to 0.77 [35].

#### 2.3.2. Liebowitz Social Anxiety Scale

The French version of the LSAS [38,39] is a 24-item scale that assesses a range of social interaction and performance situations that individuals with social phobia may fear and/or avoid. For each item, a score is provided for fear (from 0 = ‘no fear’ to 3 = ‘severe fear’) and for avoidance (from 0 = ‘no avoidance’ to 3 = ‘frequent avoidance’). The total score of the scale thus ranges from 0 to 144. Based on the work of Bouvard & Cottraux [40] and Mennin *et al*. [41], participants were considered eligible for the study if they presented an LSAS total score higher than or equal to 30 (sub-clinical social anxiety) but lower than 96 (probable clinical social anxiety).^7^ For all statistical analyses conducted, the total score of the LSAS was used. The internal consistency (Cronbach’s alpha) of the total score on the French LSAS was equal to 0.94 in the most recent study that tested its psychometric properties [38]. This scale was administered at baseline (each week), during the programme (each week) and at the three-month follow-up (see figure 2 for details).

#### 2.3.3. Self-Discrepancy Scale

The Self-Discrepancy Scale (S-DS) was developed in French by Philippot *et al.* [28] and is anchored in the self-discrepancy theory [27]. It consists of two sections: one defining the ideal self and estimating the discrepancy between the ideal self and the actual self, and one defining the socially prescribed self and estimating the discrepancy between the socially prescribed self and the actual self. In the first section, participants are first asked to generate a list of characteristics (maximum eight) that they ideally wish to have (desired traits) and a list of characteristics (maximum eight) that they ideally wish not to have (undesired traits). For each trait, participants are asked to estimate whether they possess the latter characteristics on a scale ranging from 0 to 100%. Then, participants are asked to estimate (i) the perceived gap between their ideal and actual selves (on a Likert scale from 1 = ‘I feel very near to this ideal’ to 7 = ‘I feel very far to this ideal’), and (ii) the resulting distress of this potential discrepancy (on a Likert scale from 1 = ‘I experience no distress related to this discrepancy’ to 7 = ‘I experience a strong distress related to this discrepancy’). The same procedure is repeated in the second section, which assesses the discrepancy between the actual self and the socially prescribed self. This procedure allows for computing various scores, including desired ideal trait percentage, undesired ideal trait percentage, desired prescribed trait percentage, undesired prescribed trait percentage, the gap between the actual and ideal selves (ideal gap), the distress elicited by that discrepancy (ideal distress), the gap between the actual and the socially prescribed selves (prescribed gap), and the distress elicited by that discrepancy (prescribed distress). Details concerning the computation and psychometric properties of these various scores are provided by Philippot *et al*. [28]. In the current study, the S-DS was used three times (first week of baseline, end of the programme, follow-up; see figure 3 for details), and only two indices were retained for the pre-registered hypotheses: (i) the ideal self-discrepancy (obtained by summing the ideal gap and ideal distress items), and (ii) the socially prescribed self-discrepancy (obtained by summing the prescribed gap and prescribed distress items).

#### 2.3.4. Rathus Assertiveness Scale

The French version of the Rathus Assertiveness Scale (RAS; [42]) is a 30-item self-report instrument initially designed by Rathus [43] to measure assertiveness and, more broadly, the efficiency of social skills. Each item is scored on a six-point Likert scale ranging from ‘totally true’ to ‘totally false’. In the current study, the RAS was administered three times (first week of baseline, end of the programme, follow-up; see figure 2 for details), and a global score of assertiveness/social skills was used for all statistical analyses. The internal consistency (Cronbach’s alpha) of the total score on the French RAS was found to be 0.82 [44].

#### 2.3.5. UCLA Loneliness Scale

The French version of the UCLA Loneliness Scale (UCLA-LS; [45]) is a 20-item scale designed to measure one’s subjective feelings of loneliness and social isolation. Each item is scored on a four-point Likert scale (ranging from 1 = ‘never’ to 4 = ‘often’). The internal consistency (Cronbach’s alpha) of the total score on the French UCLA-LS was found to be 0.89 [45]. For all statistical analyses conducted, the total score of the UCLA-LS was used. This scale was administered at baseline (each week), during the programme (each week) and at the three-month follow-up (see figure 2 for details).

### 2.4. Tabletop role-playing game programme

Participants played TTRPGs in the context of a *pre-established* and *structured* protocol. This programme lasts 10 weeks and is divided into 10 weekly sessions organized within three modules (see table 1 for details). Each module deploys one written role-playing scenario designed to challenge the players in game terms and involve them in a story based on maturing relationships with other characters and solving challenges by social means and investigation. These professionally designed scenarios ensure the experiment is coherent and comparable in each group. Each session lasts two hours. During our intervention, this TTRPG-based programme was delivered sequentially to four groups of five participants (see figure 2) by the same member of the research team, who is a seasoned game master, having played TTRPGs for over 30 years. The scenarios used in this programme are adapted from the free DnD introductory adventure *Lost Mine of Phandelver* (https://www.dndbeyond.com/sources/lmop). The rule system used in this programme is from the game *Chroniques Oubliées Fantasy* (https://black-book-editions.fr/catalogue.php?id=13), which is very similar to DnD, yet more straightforward to play and, therefore, particularly suitable for persons who have never played TTRPGs. Progressively, the participants are exposed to more challenging tasks, and their characters become more powerful and acquire new skills (i.e. they ‘gain levels’). At any time during the TTRPG sessions, if a participant is not feeling well regarding what is going on in the game (e.g. someone afraid of spiders in the real world must confront a giant spider in the game), they can use an ‘X card’ which signals that they want to skip the current scene. A participant is not asked why they used the ‘X card’, but they can tell the game master afterwards to avoid future incidents.

**Table 1.** TTRPG-based programme. (Note: TTRPG = tabletop role-playing games; GM = game master; NPC(s) = non-player character(s); [X/Y]: numbers reported in brackets correspond to the number of participants over the total number of participants available at a time (i.e. total number of participants minus dropouts minus participants missing a specific session) who reached, partially reached, or did not reach the objectives implemented in TTRPG programme.)

module	week	session title and synopsis	description of the session and TTRPG aspects	objectives	outcomes assessed by the GM
‘Wanderer’ module (low difficulty)	1	**Let’s get started** (one introductory session)	— welcome the participants and summarize the project and its objectives — participants are invited to present themselves briefly — introduction about TTRPGs in general — presentation of the ‘X card’ — presentation of the Medieval-Fantasy (‘medfan’) universe — introductory presentation of the rule system and character progression — character creation with the participants guiding them through the choice of race, class, characteristics and talents	— objective 1: being able to present oneself briefly to a group of unknown persons — objective 2: being able to describe the character they have created in a role-playing perspective (e.g. ‘*I am Barko Alto the gnome illusionist* […]’). The participants are invited to describe their appearance, personality, ambition, skills, etc. The description should be as comprehensive as possible and not be limited to 1−2 sentences	objective 1: — reached (enough details provided) (**20/20**) — partially reached (only very basic details like their name) (**0/20**) — not reached (not able to speak up) (**0/20**) objective 2: — reached (the participant can impersonate its character and describe comprehensively and vividly) (**20/20**) — partially reached (the participant only provides a basic and superficial description of their character) (**0/20**) — not reached (the participant is not able to present their character) (**0/20**)
	2−3	**Goblin Arrows** (two sessions) a goblins’ pack attacks the party of adventurers. They must find and explore their lair and defeat their chief	— general introduction to the world where the TTRPG will take place and the region where the participants start their adventure — the GM provides each participant with a short, personalized background element that links their character to the story (the ‘hook’) — while playing, the GM familiarizes participants with the rule system (e.g. skill checks, combat rules) — the participants must face their first group challenge (i.e. defeating a pack of goblins) — the participants must make their first group decisions (e.g. what to do after defeating the goblins: follow the goblins’ trail, fetch some help) — the participants must find the goblins’ lair (a big cave with branching paths), actively explore it, and define strategies to face and defeat various opponents, including the goblin chief — the participants gain their first level (their character is now level 2, and they increase their skills and power) — feedback 1: the GM asks the participants about their feelings and remarks about these first sessions of TTRPG playing (open feedback)	— objective 1: being able to roleplay based on one’s character ability and characteristics (e.g. race, class) — objective 2: being actively involved (≠ passive) during the encounter and/or combat scenes (e.g. combat with the goblins’ pack and the opponents encountered in the goblin cave) — objective 3: being able to actively participate in the group decision-making process (e.g. following the encounter and combat with the goblins’ pack, establishing a strategy on how to explore the cave and defeat their enemies).	objective 1: — reached (successful roleplay, being able to impersonate one’s character with sufficient realism) (**8/18**) — partially reached (basic roleplay and/or minimal use of one’s character specificity) (**10/18**) — not reached (no involvement in role-playing) (**0/18**) objective 2: — reached (actively involved in the encounter/combat scenes without the help of the GM) (**18/19**) — partially reached (involved in the encounter/combat scenes but need to be supported and/or guided by the GM and/or the other players to be active) (**1/19**) — not reached (passive attitude during the encounter/combat scenes) (**0/19**) objective 3: — reached (actively involved in the group decision-making process without the help or stimulation of the GM) (**13/18**) — partially reached (involved in the group decision-making process but need to be helped or stimulated by the GM and/or the other players) (**5/18**) — not reached (passive attitude during the group decision-making process) (**0/18**)
‘Adventurer’ module (average difficulty)	4−5	**Ruins of Thundertree** (two sessions) the party of adventurers explores the ruins of the town of Thundertree. In addition to defeating monsters and dealing with a group of evil cultists, the adventurers will encounter Reidoth the druid and Venomfang the young green dragon	— the GM introduces and provides the background for a new quest: exploring the Ruins of Thundertree — the Ruins of Thundertree, a magical and cursed place, offer a unique freedom of exploration to the participants. They can freely choose which areas to explore first and adopt various strategies to unveil all the secrets of Thundertree, enhancing their engagement and agency in the game — a new element in this module is interaction with NPCs (i.e. characters played by the DM). In the Ruins of Thundertree, the participants encounter three NPCs: (i) Reidoth the druid (friendly and collaborative); (ii) Favric the cultist (neutral and non-collaborative); and (iii) Venomfang the young green dragon (hostile and non-collaborative) — similar to the first module, the participants will face and defeat various enemies and make group decisions. However, the encounters and missions in this second module are notably more challenging, often leading to dilemmas that the participants must navigate, adding a new level of engagement and challenge to the game — the participants discover a treasure containing various items (e.g. magic artefacts and weapons, gold, equipment) and must divide it among themselves — the participants gain an additional level (their character is now level 3, and they increase their skills and power)	— objective 1: being actively involved (≠ passive) during the exploration of Thundertree and combat scenes — objective 2: being able to participate actively in the group decision-making process during the exploration of Thundertree — objective 3: being able to interact with a collaborative NPC (Reidoth the druid). The GM ensures that all participants have to interact with the druid — objective 4: being able to interact with a non-collaborative NPC (Venomfang the dragon and potentially Favric the cultist, based on their choices). The GM ensures that all participants have to interact with the NPC — objective 5: being able to participate actively in negotiation and problem-solving when dividing the treasure between the various participants	objective 1: — reached (actively involved in the exploration/combat scenes without the help of the GM) (**17/18**) — partially reached (involved in the exploration/combat scenes but need to be supported and/or guided by the GM and/or the other players to be active) (**1/18**) — not reached (passive attitude during the exploration/combat scenes) (**0/18**) objective 2: — reached (actively involved in the group decision-making process without the help or stimulation of the GM) (**18/19**) — partially reached (involved in the group decision-making process but need to be helped or stimulated by the GM and/or the other players) (**1/19**) — not reached (passive attitude during the group decision-making process) (**0/19**) objective 3: — reached (direct, proactive, and efficient interaction with the druid) (**14/18**) — partially reached (undirect, basic or DM-solicited interaction with the druid) (**4/18**) — not reached (no interaction with the druid) (**0/18**) objective 4: — reached (direct, proactive, and efficient interaction with the dragon/cultist) (**11/16**) — partially reached (undirect, basic or DM-solicited interaction with the dragon/ cultist) (**5/16**) — not reached (no interaction with the dragon/ cultist) (**0/16**) objective 5: — reached (being proactive and assertive when the treasure is divided) (**14/18**) — partially reached (being involved without being assertive enough when the treasure is divided) (**2/18**) — not reached (being passive and accepting what the other participants propose or give) (**2/18**)
	6−7	**City of Phandalin** (two sessions) the party of adventurers will freely explore the city of Phandalin, meet its inhabitants, and accomplish personal quests related to their backstory	— the GM presents the City of Phandalin, its neighbourhoods, and noteworthy inhabitants and explains to the participants that they must explore and discuss with NPCs to complete their quests — the GM introduces the concept of alignment, which corresponds to a categorization of the ethical and moral perspectives of the characters (e.g. lawful good, chaotic neutral). In this module, participants will be invited to play and role-playing according to their alignment. Only alignments that fit for ‘heroes’ will be proposed (e.g. alignments like ‘chaotic evil’ are not used) — the GM provides each participant with a personalized backstory and quest. The participants must find and interact with specific NPCs (e.g. Boker the retired paladin, Erik High-Hill the halfling innkeeper, Brocc the archmage) in the City of Phandalin. Finding good NPCs or places will involve group work. Still, for the first time, each participant must interact with a specific NPC to achieve their personal quest — the participants gain an additional level (their character is now level 4, and they increase their skills and power)	— objective 1: being able to roleplay according to their alignment — objective 2: being able to collaborate with the other participants so that each character completes their personal quest (e.g. being assertive, collaborating with other participants, making compromises) — objective 3: being able to interact individually with a specific NPC to achieve their personal quest	objective 1: — reached (roleplay coherent with the alignment and personal quest) (**11/18**) — partially reached basic roleplay not necessarily related to the alignment or personal quest) (**4/18**) — not reached (no involvement in role-playing) (**3/18**) objective 2: — reached (successful collaboration and assertiveness with the other participants) (**16/18**) — partially reached (limited collaboration and assertiveness with the other participants) (**2/18**) — not reached (passive attitude and poor collaboration with the other participants) (**0/18**) objective 3: — reached (proactive and efficient interaction with the NPC related to their personal quest) **(10/18**) — partially reached (basic or DM-solicited interaction with the NPC related to their personal quest) **(5/18**) — not reached (no interaction with the NPC related to their personal quest) (**3/18**)
‘Hero’ module (high difficulty)	8−9	**Wave Echo Cave** (two sessions) the party of adventurers will explore the Wave Echo Cave, a gigantic maze of magic and doom	— the GM introduces and provides the necessary background for a new quest: exploring the Wave Echo Cave — in this last module, the participants will again encounter NPCs, some of whom they have encountered previously. The degree of interaction will be higher as the NPCs use past decisions from the group to provoke them and stimulate debate among the participants — the specificity of this last module is that each participant is invited, each in turn, to endorse the GM role and play a unique scene corresponding to one of the rooms/areas of the Wave Echo Cave	— objective 1: being able to roleplay when it includes being in opposition to characters played by other participants — objective 2: being able to play an entire scene as the GM. The GM facilitates the process when necessary (e.g. the GM can provide participants with a series of ‘ready to play’ scripts when the participants do not want to create their scene). The participants are not forced to endorse the GM role if they do not want to	objective 1: — reached (successful resolution of conflicts, proactive to find compromise) (**18/18**) — partially reached (limited interaction, avoid conflict rather than resolve it) (**0/18**) — not reached (passive attitude, unable to deal with conflicts) (**0/18**) objective 2: — reached (the participant is able to create and play their scene as the GM) (**18/18**) — partially reached (the participant uses a script provided by the research team but is then able to endorse the GM role) (**0/18**) — not reached (the participant refuses to endorse the GM role) (**0/18**)
	10	**To be continued** (one closing session)	— if all scenes of the Wave Echo Cave have not been played (typically the scene of the fifth participant), play the remaining scene(s) — the participants are confronted with a final boss that they must confront together — the participants gain an additional level (their character is now level 5, and they increase their skills and power) — feedback 2: the participants are invited to provide open feedback about the whole experience they lived together — the GM thanks the participants for completing the programme and reminds them that a follow-up assessment will be held in three months. They receive a set of multi-sided dice as a reward — interested participants are encouraged to continue playing independently, and possibilities are discussed (e.g. participants continue to play as a group, one becomes the new GM, participants join a club)	— unless a participant has yet to play their scene in the Wave Echo Cave, the last session has no specific objective. It is about fun and feedback	not applicable

In the first module, the *Wanderer* module, the participants discover the game and create their characters (e.g. dwarf fighter, human mage, gnome rogue) under the supervision of the game master (session 1), learn and test the rules of the game (sessions 1 and 2) and play their first introductory adventure (sessions 2 and 3). This first module mobilizes low levels of social skills and assertiveness and is thus considered *low* difficulty.

In the second module, the *Adventurer* module, the participants continue their adventure by playing two scenarios managed by the game master (sessions 4 to 7). The participants must cooperate, be assertive when necessary and collectively engage to master and succeed at the scenario. Assertiveness, for example, is to be mobilized through role-played interactions between the players and non-player characters encountered in the game impersonated by the game master. Examples of these non-player characters include a friendly and collaborative druid or a non-collaborative and selfish green dragon (see table 1 for details). In the second part of this module, participants are attributed a personal backstory (specifically created to reflect the background of their character) involving a specific quest or mission. To achieve it, each participant must engage in dialogue with non-player characters (played by the game master) and potentially negotiate (and thus be assertive) with the other participants. These personal quests are used to ensure that each participant is sufficiently exposed. This second module mobilizes a medium level of social skills and assertiveness and is thus considered *moderate* difficulty.

Finally, in the third module (sessions 8−10), the *Hero* module, the participants are invited to take on the role of the game master to propose a short scene themselves (with the help of the game master from the research team, when necessary; see table 1 for details). The adventure takes place in the *Wave Echo Cave*, a magical maze with dozens of caves and areas involving various dangers and events. This final module engages the players in new ways, as each participant must take control of the game, tell the story and guide the other participants into an adventure. To reach this objective, each participant successively takes on the role of the game master and designs the scene they want to play with the other participants. The game master from the research team supports this transition from player to game master through light tutoring and providing ideas and tips when necessary. This third module mobilizes higher levels of social skills and assertiveness, is thus considered *high* difficulty. The final session (session 10) includes a debriefing; and qualitative feedback about the experiment was collected.^8^

At the end of each session, the game master (who did not have access to the results of the psychological assessments) coded for each participant whether the session objectives were achieved, partially achieved or not achieved (see table 1 for details). In this study, we considered objective achievement assessed by the game master as an indicator of feasibility.^9^ Indeed, it was deemed important that a large proportion of participants successfully attain the progressively more difficult objectives implemented in the three modules composing the TTRPG programme, both from a motivational (i.e. self-efficacy enhancement, positive reinforcement) and engagement (i.e. as participant’s commitment is a good indicator of treatment adherence/therapeutic alliance) perspective.

### 2.5. Data analytic strategy

Because no gold standard exists for statistical analyses in single-case studies and because the different analyses available focus on different data features, all of which have advantages and flaws [34], we used a series of complementary steps to analyse the data. This approach allowed us to use several effect size measures that can be compared to assess the consistency of the results. First, non-overlap Tau-U indices were used to quantify the proportion of data points in the intervention phase (B) that had improved with respect to the baseline phase (A) measurements while controlling for baseline trend [46,47]. This measure thus provided separate values for each (AB) comparison individually, with positive values indicating an improvement with respect to the baseline and negative values indicating a deterioration. Second, we capitalized on an index that aims to determine whether changes at the end of the TTRPG-based programme and the three-month follow-up could be considered significant [48]. This method was performed by considering the participants for all outcomes separately. The first reported scores represent the baseline scores, the second reported scores represent the post-intervention scores, and the third reported scores represent the three-month follow-up scores. The reliable change index/clinically significant change calculations were obtained from means, standard deviations and reliability coefficients reported in the published articles of the various measurement tools used (IGDT-10; [35] LSAS; [38] UCLA-LS; [45] RAS; [42] S-DS; [28]). Participants moving reliably into the functional distribution are considered recovered, improved if they have made a reliable change but remain in the dysfunctional population, unchanged if they have not made a reliable change, and deteriorated if they have reliably worsened. The terms ‘recovered’ and ‘improved’ are used to adhere to the labels proposed by Jacobson & Truax [48], even if the present study was conducted with non-clinical participants. Third, between-cases standardized mean differences (BC-SMD) were used to quantify the difference between the baseline and intervention phases’ scores of each primary outcome (see below), providing overall quantification across all study participants (similar to Cohen’s *d*) while dealing with autocorrelation [49]. The following study outcomes are expected:

(i) *primary outcome 1*: a reduction of self-reported time spent playing video games (average number of hours per day) at the end of the TTRPG-based programme (P1A);^10^
(ii) *primary outcome 2*: a reduction of gaming disorder symptoms (assessed by the IGDT-10) at the end of the TTRPG-based programme (P2A) and the three-month follow-up (P2B);
(iii) *primary outcome 3*: a reduction of social anxiety symptoms (assessed by the LSAS) at the end of the TTRPG-based programme (P3A) and the three-month follow-up (P3B);
(iv) *secondary outcome 1*: an increase in assertiveness and self-perceived social skills (assessed by the RAS) at the end of the TTRPG-based programme (S1A) and the three-month follow-up (S1B);
(v) *secondary outcome 2*: an improvement in self-concept reflected by a lower ideal self-discrepancy and a lower socially prescribed self-discrepancy (assessed by the S-DS) at the end of the TTRPG-based programme (S2A) and the three-month follow-up (S2B); and
(vi) *secondary outcome 3*: a decrease in loneliness (as indexed by the UCLA-LS) at the end of the TTRPG-based programme (S3A) and the three-month follow-up (S3B).

Participants with missing data would have been omitted from the analyses if the number of measurement points per phase (baseline and intervention) was lower than three, as three measurement points per phase are considered the minimum standard to reach in a single-case methodology [34]. This situation did not occur in the present study, and all participants who had not dropped out before the end of the intervention were kept for the analyses (*n* = 18).

The data analytic plan was pre-registered upon in-principle acceptance of the stage 1 registered report (see https://osf.io/hzyva). Tau-U non-overlap indices and between-case standardized mean differences were computed on *R* 4.4.0 [50] with the following packages:

(i) *scdhlm* package version 0.7.3 ([51]; https://CRAN.R-project.org/package=scdhlm); and
(ii) *SingleCaseES* package version 0.7.2 ([52]; https://CRAN.R-project.org/package=SingleCaseES).

## 3. Results

### 3.1. Feasibility of the tabletop role-playing game programme

A dual approach was used to document the feasibility of the TTRPG programme. The first approach is based on actual involvement in the TTRPG programme (i.e. number of dropouts, number of TTRPG sessions attended, number of completed psychometric assessments). The second approach is based on objective achievement as assessed by the game master (see table 1 for a comprehensive list of objectives per module and session for the TTRPG programme and how they can be reached, partially reached or not reached).

Two participants dropped out of the TTRPG programme (see figure 3). Both participants reported having dropped out because the TTRPG sessions were too stressful for them. One participant dropped out directly after the first session, while the other dropped out after session 4, corresponding to the beginning of the second module in which players are progressively confronted with more demanding objectives (e.g. interacting with non-playing characters, negotiating with other players). Both participants were individually contacted by the research team and oriented towards an individual psychological treatment.

Among the 18 completers, most (10 out of 18) participated in all 10 sessions, while some (6 out of 18) missed a single session. Only two participants missed more than one session: one participant missed two consecutive sessions, while the other missed three sessions (sessions 4, 6 and 10). All missed sessions were justified by the participants. The most frequent reasons mentioned by the participants included being ill, preparing for exams, forgetting an appointment or taking time off. All in all, these data indicated a high implication and participation in the TTRPG programme by our participants. Data related to the number of sessions attended by participants can be retrieved from the Open Science Framework (see https://osf.io/fqj2e).

We were also interested in the participants’ ability to complete the weekly online psychometric assessment (during the baseline and the intervention) and the three-month follow-up. Completion rates are reported in figure 3. All 20 participants completed the three measurement points of the baseline. Among the 18 completers, 17 completed the 10 weekly psychometric assessments and the three-month follow-up. One participant completed the 10 weekly psychometric assessments but not the three-month follow-up.

In this study, a series of 15 objectives of progressive difficulty were implemented in the three modules of the TTRPG programme (see table 1 for a detailed account of these objectives). The game master assessed the attainment of each participant. As explained before, we reasoned that for our programme to be feasible, most participants should succeed (or partially succeed) in these objectives. As depicted in figure 1, most participants reached all but one of these objectives (i.e. 14 out of 15). A notable exception is the objective ‘being able to roleplay based on one’s character ability and characteristics’ (session 2−3, module 1), for which a majority of participants partially succeeded (i.e. they only showed basic roleplay or minimal use of one’s character specificity). This can be explained by the fact that this was the first time in our programme that participants were instructed to proactively participate in role-playing, which is an even more demanding task for persons with social anxiety. All objectives implemented in module 1 (‘the Wanderer module’) were reached or partially reached by the participants. A minority of participants did not reach all objectives implemented in module 2 (‘Adventurer module’) and module 3 (‘Hero module’). The most challenging objectives were not reached by a maximum of three participants (objectives 1 and 3 of sessions 6 and 7, module 3, see table 1 for details). Overall, the objectives that were the hardest to achieve for our participants were those involving complex role-playing (e.g. role-playing according to a specific situation or ‘alignment’) or assertiveness vis-à-vis other players (e.g. being able to participate actively in negotiation and problem-solving when dividing a treasure between the various characters played by the participants). All individual participant data regarding objective attainment are available on the Open Science Framework at https://osf.io/sz6p8.

### 3.2. Efficacy of the tabletop role-playing game programme

#### 3.2.1. Baseline (A) to intervention (B) comparisons using non-overlap Tau-U indices

The results obtained using non-overlap Tau-U indices between the baseline (A) and the intervention (B) phases indicated a large inter-individual heterogeneity, with symptoms decreasing to various degrees among participants (see table 2). Avoidance of social situations symptoms decreased to various degrees in 16 participants (88.89% of the sample), 13 of whom showed a Tau-U between 1.00 and 0.60. Fear of social situations symptoms decreased in 17 participants (94.44%), 10 of whom showed a Tau-U between 1.00 and 0.60. Gaming disorder symptoms decreased in 15 participants (83.33%), five of whom showed a Tau-U between 1.00 and 0.60. Time spent gaming decreased in 10 participants (55.56%), two of whom showed a Tau-U between 1.00 and 0.60. Perceived loneliness decreased in 11 participants (61.1%), six of whom showed a Tau-U between 1.00 and 0.60.

**Table 2.** Non-overlap Tau-U indices. (Note: *n* = 18. A positive value indicates an improvement with respect to the baseline, whereas a negative value indicates a deterioration.)

variable	Tau-U interval	Tau-U between 1.00 and 0.60 *n* (%)	Tau-U between 0.59 and 0.01 *n* (%)	Tau-U of 0 or less *n* (%)
gaming disorder symptoms (IGDT−10)	[−0.57, 0.87]	5 (27.78)	10 (55.56)	3 (16.67)
fear of social situations symptoms (LSAS)	[−0.40, 1.00]	10 (55.56)	7 (38.89)	1 (5.56)
avoidance of social situations symptoms (LSAS)	[−0.83, 1.00]	13 (72.22)	3 (16.67)	2 (11.11)
perceived loneliness (UCLA-LS)	[−0.90, 0.97]	6 (33.33)	5 (27.78)	7 (38.89)
time spent gaming (number of hours per week)	[−0.67, 0.67]	2 (11.11)	8 (44.44)	8 (44.44)

For a few participants (between one to three participants for gaming disorder symptoms and social anxiety symptoms; see table 2), Tau-U indices were negative, suggesting that symptoms tended to worsen during the intervention in some cases. With respect to perceived loneliness, four participants showed Tau-U indices ranging from −0.70 to −0.90, indicating that their perceived loneliness increased during the intervention (B) phase compared with the baseline (A) phase. Similarly, for time spent gaming, six participants showed a negative Tau-U ranging from −0.03 to −0.67. Of note, individual results are available on the Open Science Framework at https://osf.io/3pgt7/.

#### 3.2.2. Baseline (A) to intervention (B) and follow-up (C) comparison using reliable and significant change indices on primary outcomes

Table 3 presents a synthesis of results obtained using reliable and significant change indices according to Jacobson & Truax’s [48] method. As a reminder, the terms ‘recovered’ and ‘improved’ are used to adhere to the labels proposed by Jacobson & Truax [48], even though our study was conducted with non-clinical participants.

**Table 3.** Reliable and significant change indices (primary outcomes).

phases	variable	recovered *n* (%)	improved *n* (%)	unchanged *n* (%)	deteriorated *n* (%)
**baseline (A)–intervention (B**) (*n* = 18)	gaming disorder symptoms (IGDT−10)	6 (33.33)	0 (0)	12 (66.67)	0 (0)
	fear of social situations symptoms (LSAS)	4 (22.22)	3 (16.67)	11 (61.11)	0 (0)
	avoidance of social situations symptoms (LSAS)	2 (11.11)	4 (22.22)	12 (66.67)	0 (0)
**baseline (A)–three-month follow-up (C**) (*n* = 17)	gaming disorder symptoms (IGDT−10)	1 (5.88)	0 (0)	15 (88.24)	1 (5.88)
	fear of social situations symptoms (LSAS)	4 (23.53)	1 (5.88)	12 (70.59)	0 (0)
	avoidance of social situations symptoms (LSAS)	2 (11.76)	2 (11.76)	13 (76.47)	0 (0)

Results indicated positive short-term effects—at the end of the intervention (B) phase—on gaming disorder symptoms for 33.3% of participants, with only one participant maintaining a recovery at the three-month follow-up (C) phase; only one reported that their symptoms deteriorated at the three-month follow-up. Regarding social anxiety symptoms, both fear and avoidance of social situations symptoms improved in the short term (B) for 39% and 33% of the sample, respectively; this improvement was maintained for 29% and 23% of the participants, respectively, at the three-month follow-up (C) phase. Three persons recovered in the short-term (B) and at the three-month follow-up (C) phase on the fear scale, whereas one person recovered in the short-term (B) and at the three-month follow-up (C) on the avoidance scale. Note that time spent gaming was not presented in these additional analyses because, without existing psychometric data on this variable (notably reliability), the reliable and significant change indices could not be estimated with Jacobson & Truax’s [48] method. Of note, individual results are available on the Open Science Framework at https://osf.io/3pgt7/.

#### 3.2.3. Between case-standardized mean differences between baseline (A) and intervention (B): a general effect size for each primary outcome

The results obtained using BC-SMD between the baseline (A) phase and the intervention (B) phase showed that scores on all the variables examined decreased during the intervention (B) phase compared with the baseline (A) phase across the entire sample (table 4).

**Table 4.** Between case-standardized mean differences (BC-SMD). (Note: *n* = 18. The lower the BC-SMD, the better the improvement.)

variable	BC-SMD [95% CI]	s.e*.*
gaming disorder symptoms (IGDT−10)	−0.39 [-0.73, −0.04]	0.17
fear of social situations symptoms (LSAS)	−0.20 [-0.44, 0.03]	0.11
avoidance of social situations symptoms (LSAS)	−0.47 [-0.76, −0.19]	0.14
perceived loneliness (UCLA-LS)	−0.38 [-0.85, 0.08]	0.23
time spent gaming (number of hours per week)	−0.15 [-0.44, 0.15]	0.14

Overall, these results are consistent with those obtained via the Tau-U (see table 2) insofar as they indicated that the avoidance of social situations symptoms constituted the variable that benefited the most from the intervention, both at the group (BC-SMD) and individual (Tau-U) level, whereas time spent gaming was the least positively affected.

#### 3.2.4. Baseline (A) to intervention (B) and follow-up (C) comparison using the reliable and significant change level on secondary outcomes

Secondary outcomes considered included assertiveness, self-concept and perceived loneliness. As a reminder, the terms ‘recovered’ and ‘improved’ are used to adhere to the labels proposed by Jacobson & Truax [48], even though our study was conducted with non-clinical participants. With respect to assertiveness (table 5), the results obtained using the reliable and significant change indices indicate an overall absence of change in this variable. However, a deterioration in assertiveness in the short-term (B) was reported for one participant, but with a return to baseline (A) phase level at the three-month follow-up (C) phase, whereas a deterioration was observed for one participant and recovery for one other participant at the three-month follow-up (C) phase. Similarly, regarding self-discrepancy (S-DS), most participants did not change, regardless of the phases compared. However, some persons reported a recovery in the short-term between their perceived self and their ideal self or on the distress associated with the gap between their perceived and their ideal self with a return to the baseline (A) phase level at the three-month follow-up (C) phase. Regarding perceived loneliness, 33% of participants improved their score, 56% did not change in the short term and 11% decreased their score in the short term (B). One person recovered in the short-term (B) and at the three-month follow-up (C) phase.

**Table 5.** Reliable and significant change indices (secondary outcomes).

phases	variable	recovered *n* (%)	improved *n* (%)	unchanged *n* (%)	deteriorated *n* (%)
**baseline (A)–intervention (B**) (*n* = 18)	assertiveness (RAS)	0 (0)	0 (0)	17 (94.44)	1 (5.56)
	ideal gap (S-DS)	1 (5.56)	0 (0)	17 (94.44)	0 (0)
	ideal distress (S-DS)	2 (11.11)	0 (0)	16 (88.89)	0 (0)
	prescribed gap (S-DS)	0 (0)	0 (0)	18 (100)	0 (0)
	prescribed distress (S-DS)	0 (0)	0 (0)	18 (100)	0 (0)
	perceived loneliness (UCLA-LS)	4 (22.22)	2 (11.11)	10 (55.56)	2 (11.11)
**baseline (A)–three-month follow-up (C**) (*n* = 17)	assertiveness (RAS)	1 (5.88)	0 (0)	15 (88.24)	1 (5.88)
	ideal gap (S-DS)	0 (0)	0 (0)	15 (88.24)	2 (11.76)
	ideal distress (S-DS)	2 (11.76)	0 (0)	15 (88.24)	0 (0)
	prescribed gap (S-DS)	0 (0)	0 (0)	17 (100)	0 (0)
	prescribed distress (S-DS)	1 (5.88)	0 (0)	16 (94.12)	0 (0)
	perceived loneliness (UCLA-LS)	1 (5.88)	2 (11.76)	13 (76.47)	1 (5.88)

Of note, a comprehensive case report illustrating the effect of our 10-week pre-established and structured protocol is available on the Open Science Framework at https://osf.io/u4tpj.

## 4. Discussion

This registered exploratory pilot aimed to test the feasibility and initial efficacy of a 10-session structured protocol in which socially anxious online gamers are exposed to offline social skills and real-life social interactions while playing an offline TTRPG. In terms of feasibility, we observed that most participants completed the programme (18 out of the 20 participants completed the 10-week intervention) and were engaged in terms of attendance (of the 18 completers, 10 missed no sessions, six missed a single session and two missed more than a single session) and completion rates of weekly psychometric assessments (all weekly baseline and intervention phases assessments were completed, and one participant did not complete the three-month follow-up phase assessment). Moreover, participants were largely able to achieve the progressively more difficult objectives implemented in the TTRPG programme. Taken together, these data suggest a high level of motivation and involvement in our programme. A potential explanation for these very encouraging results is that TTRPGs share many features with video games (especially MMORPGs and online RPGs) and similarly allow for fulfilling basic individual needs such as relatedness, autonomy and competence. Of course, we cannot rule out the possibility that other aspects (e.g. financial compensation, relationship of trust with the game master, specific group dynamics, conscientiousness of the participants) may have influenced the motivation and engagement of the participants. The observed results are encouraging and suggest that TTRPG-based intervention programmes may reduce social anxiety and gaming disorder symptoms.

In terms of initial efficacy, various degrees of improvement were identified regarding primary study outcomes (e.g. gaming disorder symptoms, fear and avoidance of social situations) when quantifying intervention efficacy across all participants. Crucially, single-case analyses showed that our TTRPG programme was very efficient for a subgroup of participants, while its effect was more limited or null/negative for a few participants (see table 2 for details). In this sense, our study confirms the soundness of single-case analyses for testing treatment efficacy, which in the current case allowed us to emphasize the important heterogeneity of participants’ responses to the intervention. In terms of reliable clinical change after the intervention, the effect of the intervention was less pronounced (i.e. fewer participants benefited from our programme) than what was observed with Tau-U analyses. However, this is not necessarily surprising as this pilot was conducted on a non-clinical sample. Three-month follow-up analyses suggest that our TTRPG programme has a lasting effect on social anxiety symptoms (in terms of recovery and improvement) but not on gaming disorder symptoms (most recoveries were not maintained at the three-month follow-up). It is, however, worth noting that most previous studies which tested the effect of TTRPG-mediated psychological interventions did not include a follow-up (for scoping reviews of the matter, see Arenas *et al.* [18] and Yuliawati *et al.* [53], which is also the case of most studies that tested the efficacy of traditional psychological treatments (e.g. cognitive behaviour therapy) in persons diagnosed with gaming disorder (for systematic reviews, see King *et al.* [13] and Zajac *et al.* [54]). The current state of the literature thus hinders comparison of the long-lasting effect of our exploratory registered pilot with previous work that tested the effect of TTRPG-mediated interventions on mental health or traditional psychological treatment on problematic gamers. All in all, the results of the present pilot are promising and pave the way for future studies in clinical samples of socially anxious gamers. However, the question remains as to whether a clinical population would require adjustments to the programme (e.g. adjusting the difficulty of the game objectives or the number of participants per group).

Primary outcomes targeted in this exploratory pilot were reductions in time spent playing video games (P1A), gaming disorder symptoms (P2A and P2B) and social anxiety symptoms (P3A and P3B). Self-reported time spent gaming decreased during the intervention phase compared with the baseline phase for a majority of the sample (10 out of 18 cases), whereas for a significant portion of the sample (8 out of 18 cases), no change or even an increase in self-reported time spent gaming was observed (see table 2). It is important to keep in mind that time spent gaming should not necessarily be regarded as an indicator of problematic gaming and that many gamers can be highly involved in gaming (i.e. playing several hours a day) without experiencing any kind of negative consequences or functional impairment [32,55,56]. Yet, in the present study, over-involvement in gaming was conceptualized as a compensatory process [7,57], and it might be speculated that some of our participants reduced their time spent gaming because they fulfilled their unmet need for social affiliation/contact during the period in which the intervention took place (e.g. through the TTRPG sessions or through other social activities they have started concomitantly to being involved in our study). We should, however, remain cautious when interpreting results obtained with self-reported items assessing time spent in digital media use, as they rarely provide an accurate reflection of actual (or logged) media use (for a systematic review and meta-analysis, see Parry *et al.* [58]). Of note, and as explained in the ‘Methods’ section, it was unfortunately not possible for methodological reasons to test the reduction in time spent gaming at the three-month follow-up. Gaming disorder symptoms diminished post-intervention for most participants (15 out of 18 cases).^11^ Additional analyses suggest that one-third of our participants (6 out of 18 cases) presented with what can be considered a clinically relevant recovery from their gaming disorder symptoms after the intervention, although only one case maintained this recovery at the three-month follow-up (see table 3). Regarding social anxiety symptoms, fear of social situations was reduced for almost all participants (17 out of 18 cases), which was also the case for the avoidance of social situations (16 out of 18 cases). The magnitude of the decrease was the most pronounced for the avoidance of social situations after the intervention phase compared with the baseline phase, according to the BC-SMD analyses. It cannot be ruled out that the result regarding avoidance of social situations has been at least partly influenced by the fact that the participants might have considered the TTRPG sessions *per se* as exposure to social situations. Additional analyses suggested that approximately one-third of our participants (7 out of 18 cases for fear of social situations, 6 out of 18 cases for avoidance of social situations) presented with a clinically relevant recovery or improvement after the intervention and that most of them maintained this recovery or improvement at the three-month follow-up (see table 3). Of note, the terms ‘recovered’ and ‘improved’ are used to adhere to the labels proposed by Jacobson & Truax [48] to define clinically relevant changes in psychotherapy research, even though our study was conducted with non-clinical participants.

Taken together, these results suggest that our TTRPG programme may improve gaming disorder and social anxiety symptoms, at least in the short term. It is not surprising that the impact of our intervention was more pronounced on social anxiety symptoms, as our TTRPG programme includes several ingredients that are implemented in evidence-based psychological treatment for social anxiety, such as progressive exposure to social situations or exercises mobilizing social skills and assertiveness. This study is, to our knowledge, the first to show that TTRPG-mediated interventions have the potential to reduce gaming disorder symptoms, thus answering recent calls to develop and validate new psychological treatments for persons exhibiting problematic gaming habits [3,4]. Moreover, the results obtained in our registered pilot reproduce, by capitalizing on another methodology (i.e. a multiple single-case approach), the findings from previous work that showed that TTRPG-mediated interventions may mitigate social anxiety symptoms [23].

Secondary outcomes targeted in this exploratory pilot were an increase in assertiveness and self-perceived social skills (S1A and S1B), an improvement in self-concept reflected by a lower ideal self-discrepancy and a lower socially prescribed self-discrepancy (S2A and S2B), and a decrease in perceived loneliness (S3A and S3B). Two of these secondary outcomes (assertiveness/self-perceived social skills and self-concept) were only assessed three times during the study (first week of the baseline, last week of the intervention, and at the three-month follow-up), implying that the analyses conducted were limited to the clinically relevant change index at post-intervention and follow-up. The analyses revealed that neither assertiveness/self-perceived social skills nor self-concept-related constructs (e.g. the discrepancy between perceived self and ideal self) were affected by our TTRPG programme. In this study, these psychological constructs were assessed to identify, from a process-based perspective [59,60], the psychological mechanisms potentially involved in the symptoms displayed by the participants. First, although our intervention successfully mitigated fear and avoidance of social situations, it did not improve assertiveness/social skills *per se*. These results could suggest that the improvement of social anxiety symptoms not necessary implies an actual improvement in assertiveness/social skills. As previous studies only found TTRPG interventions to improve social skills in clinical populations (e.g. autism spectrum disorder, see Katō [25]), further studies should be conducted in clinical and non-clinical samples, using different and/or more comprehensive assessment approaches. Second, our intervention did not appear to modify self-concepts as assessed by the S-DS [60]. This is not necessarily surprising, as self-concepts such as the ideal self, the perceived self or the socially prescribed self [27] are deeply rooted psychological processes influenced by multi-determined factors such as personality, life events, education or personal values. In this sense, our 10-week group-based TTRPG programme is probably not sufficient—for example, compared with an individual psychotherapeutic setting—to alter such long-lasting and relatively stable psychological dimensions. This contrasts with a recent study (that was not available at the time we conceptualized the present study) which showed that an eight-week TTRPG-based intervention (using DnD) was able to improve self-reported self-esteem [61]. However, caution is required when comparing our study and the study by Merrick *et al*. [61], as the instruments used to assess self-concepts in each study are not comparable and measure different constructs. Finally, perceived loneliness diminished post-intervention for some participants (11 out of 18 cases, see table 2). Yet, our results also showed that, for a large portion of the participants (7 out of 18 cases), perceived loneliness either remained unchanged or even deteriorated. Additional analyses showed that some participants (6 out of 18 cases) displayed a clinically relevant recovery or improvement from their perceived loneliness after the intervention, although only a minority (3 out of 18 cases) maintained this recovery or improvement at the three-month follow-up (see table 5). Again, the terms ‘recovered’ and ‘improved’ are used to adhere to the labels proposed by Jacobson & Truax [48] to define clinically relevant changes in psychotherapy research, even though our study was conducted with non-clinical participants. The results suggest that our TTRPG-based programme temporarily improved perceived loneliness in some of our participants. Yet, we also observed opposite findings where a substantial part of our participants still experienced loneliness or even increased their perceived loneliness during the intervention. A possible explanation for this surprising pattern of results could be that, for some participants, regularly playing TTRPGs with other people paradoxically magnifies their feeling of being lonely when not playing. We also observed that, for some participants, perceived loneliness decreased after the first few sessions, then began to increase—sometimes dramatically—as they approached the last sessions of the TTRPG programme and realized that their weekly social sharing and playing event would soon come to an end. This phenomenon is well illustrated in a comprehensive case analysis of one of our participants, available on the Open Science Framework at https://osf.io/u4tpj.

The present study has several limitations. First, the measurement points in the baseline were identical for each group (three measurement points per baseline), while international standards for defining robust experimental single-case methodology suggest adopting different numbers of measurement points between groups or participants and staggering the onset of the intervention across groups as defined in a concurrent multiple baseline design [34]. As acknowledged in the methods section, we initially planned baselines with three to six measurement points per group, but we had to switch to a similar three measurement point for all the groups for practical reasons. Yet, and importantly, three measurement points were sufficient to perform all planned analyses, although it implied that our design must be considered quasi-experimental instead of fully experimental. Second, we only had a single measurement point for the three-month follow-up, which hindered us from computing several key analyses with respect to this follow-up (e.g. non-overlap Tau-U indices, BC-SMD). When elaborating our research protocol, we reasoned that it would be impractical to ask participants to fill out multiple follow-up assessments and thus opted for a single follow-up assessment. However, based on the completion rate of psychometric assessments in the present study (see ‘Feasibility of the tabletop role-playing game programme’ section for more details), further studies could try to implement a follow-up phase with multiple measurement points. Third, the analyses conducted cannot account for potential complex group effects and dynamics (e.g. some groups might be more or less supportive, and affinities between players could differ across groups). This issue might be at least partly counter-balanced by the fact that the same seasoned game master administered the programme to all four groups. Yet, future studies should investigate whether group-related variables (e.g. dynamics and relationships between players) affect the outcome of TTRPG-based interventions. Fourth, the analyses did not consider that some participants could have missed one or more of the TTRPG sessions. Nevertheless, most completers participated in all 10 sessions, and only two participants missed more than one session, which implies that this issue probably had no impact on the present study. Eventually, it was not possible for practical reasons (i.e. time available during the TTRPG sessions) to collect qualitative data about the study as initially planned. It would indeed have been interesting to ask participants what aspects (or modules) of the programme they liked or disliked, whether they found the programme helpful (or not), or what reasons motivated them to complete it. We could also have inquired whether certain parts of the programme were more difficult for them and why, or whether they triggered negative emotions such as anxiety. Participants could also be asked if they can identify areas or skills that they feel were improved by the programme. These topics should be investigated in future studies testing the therapeutic potential of TTRPG-mediated psychological interventions using a qualitative approach.

## 5. Conclusions

The current pilot study demonstrated that a 10-week structured TTRPG-based intervention is feasible and might be effective in reducing symptoms in a sample of sub-clinical socially anxious gamers. Furthermore, and crucially, the present study is, to our knowledge, the first quantitative study of the therapeutic use of TTRPG to meet all the canons of open science, from pre-registration of the study design and research questions to open data and materials. Indeed, we described in detail the content of all TTRPG sessions and used open-access game scenarios to promote the reproducibility of our research findings. In this sense, we responded to the call for more robust and well-designed empirical studies on the application of TTRPGs in mental health [18,53]. Although our findings must be reproduced in a clinical sample of anxious gamers, the present registered exploratory pilot is highly encouraging and calls for the development of TTRPG-mediated interventions in mental health. Moreover, this work also paves the way for non-medical psychological treatments for individuals presenting problematic engagement in video games.

## Data Availability

All code, data, materials and preprints related to the present registered report are stored on European servers based in Frankfurt (Germany) and publicly shared under a CC-By Attribution 4.0 International license on the Open Science Framework.

## Footnotes

^1^
This approach was decided upon in the stage 1 registered report. However, this situation did not arise.

^2^
We reasoned that prior involvement in TTRPGs might have influenced some key psychological factors assessed in the study (e.g. social skills, self-concepts, loneliness or social anxiety symptoms) and thus would have constituted a confounding factor. Also, we considered it important that all participants included in the study undergo comparable and progressive exposure to TTRPGs. Eventually, mixing participants with and without prior experience with TTRPGs (or with different levels of prior experience with TTRPGs) would have resulted in unbalanced situations between participants (potentially more accessible for some participants and more complex for others, depending on their prior experience with TTRPGs).

^3^
Initially, potentially eligible participants scoring five or more gaming disorder criteria based on the IGDT-10 were planned to undergo an additional assessment by a clinical psychologist to verify if they endorse diagnostic requirements of gaming disorder as defined in ICD-11 (ICD-11 Code 6C51.0 Gaming Disorder, predominantly online) and exclude them from the study. We finally decided to skip this additional appointment, and participants with a possible gaming disorder based on a score of five or more on the IGDT-10 (*n* = 13; see figure 3 for the study flowchart) were directly excluded from the study and addressed to the Center for Excessive Gambling at the Lausanne University Hospital.

^4^
This did not occur in the present study (see figure 3).

^5^
Initially, it was planned to maximize heterogeneity within groups regarding gender, age and education level. However, this was eventually not feasible, so we created groups based on participant availability and ensured that none of the participants in the same group knew each other.

^6^
We initially planned to have baselines of three to six measurement points (i.e. different number of measurement points for each group) with the onset of the experimental phase randomly determined for each group (group 1 starts after a three-week baseline, group 2 starts after a four-week baseline, etc.). This methodological approach corresponds to international standards for defining an experimental single-case methodology with enough data points per phase. However, we had to adjust our design to guarantee the feasibility of the study. The reason is that the recruitment process took longer than anticipated, and we needed to finish the 10-week intervention phase before the beginning of the holiday season in Switzerland (early July) to avoid massive dropouts at the end of the study. Accordingly, we applied a similar three-week baseline for each group, implying that our design became quasi-experimental instead of experimental [34].

^7^
We initially fixed a minimum eligibility score higher than or equal to 56 for the LSAS based on the work of Bouvard & Cottraux [40] on the French version of the LSAS. Yet, as indicated in our responses to reviewers in the stage 1 registered report (available on the *PCI RR* website at https://rr.peercommunityin.org/articles/rec?id=399), we anticipated that the cut-off of 56 might be too high, which was confirmed during the initial steps of the recruitment process. Therefore, we adjusted the inclusion criteria and adopted the laxer cut-off of 30 proposed by Mennin *et al*. [41].

^8^
It was initially planned to collect high-quality qualitative data during this final session. Unfortunately, this was finally not possible for practical reasons. Indeed, there was not enough time in session 10 to implement this data collection. Instead, the game master conducted informal feedback during the debriefing of the last session; however, data were not collected in a structured way nor recorded, hindering the possibility of conducting qualitative analyses. To remedy this issue, we implemented a qualitative question in the last online assessment; however, most participants either did not answer or provided very short, non-comprehensive answers. These data were thus not analysed nor stored on the Open Science Framework.

^9^
We initially planned to conduct exploratory non-pre-registered analyses using game-master assessments of objective achievement. Yet, we finally abandoned this idea and only used these assessments to support the feasibility of our TTRPG programme. The proportion of achieved, partially achieved and non-achieved objectives is reported in table 1, and the raw data are available on the Open Science Framework at https://osf.io/65w3j.

^10^
As explained in the Data analytic strategy’ section, follow-up analyses were based on reliable change index/clinically significant change indices estimated with the method of Jacobson & Truax [48]. This method requires psychometric data (notably reliability), which were not available for self-reported time spent gaming (this variable was assessed with a single custom item). As our protocol planned only one measurement point at the follow-up phase, it was not possible to apply other types of single-case analyses, as they would have required at least three measurement points. This issue was not anticipated by the research team, and contrary to what was pre-registered in the stage 1 registered report, we did not test a reduction in time spent gaming at the three-month follow-up.

^11^
Following a request made by the *PCI RR* recommender concerning the stage 2 registered report, we added to the Open Science Framework a table reporting the proportion of IGDT-10 items endorsed by each participant before the intervention, after the intervention and at follow-up (https://osf.io/2q658).

## Authors' Notes

Joël Billieux submitted a grant application and obtained the research funds necessary to conduct the study (including a one-year 20% research associate position for Jonathan Bloch and a six-month 10% research associate position for Lucien Rochat). Yasser Khazaal, Olivier Simon, Jonathan Bloch, Andreas Lieberoth, Marc Malmdorf Andersen, and Daniel King participated in conceptualizing and writing the grant application, which included operationalizing the study hypotheses and selecting assessment instruments. Joël Billieux wrote the first draft of the Stage 1 Registered Report in collaboration with Lucien Rochat (for the “Data analytic strategy” section) and Charlotte Eben (for open science-related aspects). All other authors reviewed and edited the Stage 1 Registered Report and approved its final version. Joël Billieux and Jonathan Bloch managed the various steps leading to the ethical approval of the study by the Cantonal Commission on Ethics in Human Research (CER-VD). Loïs Fournier supervised the design and implementation of the online surveys used in the various steps of the study (eligibility screening, baseline, during the intervention, and follow-up psychological assessments). Joël Billieux, Loïs Fournier, and Iliyana Georgieva monitored the recruitment process. Loïs Fournier and Iliyana Georgieva included the eligible participants in the study, collected the informed consent forms, and distributed the participants among the various groups. Joël Billieux provided the necessary data to the person in charge of the external monitoring. Jonathan Bloch designed the various modules of the 10-week TTRPG-based program under the supervision of Joël Billieux. Jonathan Bloch administered – as the game master – the TTRPG program to the four groups of participants. Olivier Simon and Yasser Khazaal coordinated and supervised the invitations and medical appointments at the Center for Excessive Gambling (CHUV) proposed to the participants excluded from the study for presenting a possible gaming disorder. Yasser Khazaal personally contacted the participant who had been excluded during the pre-intervention individual interview and the two participants who had dropped out during the intervention (see Figure 3 for the study flowchart) and oriented them towards individual psychological treatment. Loïs Fournier uploaded all code, data, materials, and preprints on the Open Science Framework. Charlotte Eben served as the open-science co-pilot of Loïs Fournier, helping with the pre-registration and data-sharing principles. Lucien Rochat conducted the statistical analyses. Loïs Fournier curated the data and verified the reproducibility of the code for the statistical analyses. Joël Billieux wrote the first draft of the Stage 2 Registered Report in collaboration with Loïs Fournier and Lucien Rochat. All other authors reviewed and edited the Stage 2 Registered Report and approved its final version.
